# Circadian changes and sex-related differences in fetal heart rate parameters

**DOI:** 10.1186/s40748-016-0037-6

**Published:** 2016-09-02

**Authors:** Habiba Kapaya, Fiona Broughton Pipkin, Barrie Hayes-Gill, Pamela V. Loughna

**Affiliations:** 1 Division of Child Health, Obstetrics and Gynaecology, School of Medicine, University of Nottingham, Nottingham, NG7 2RD UK; 2 Electrical Systems and Optics Research Division, University of Nottingham, University Park, Nottingham, NG7 2RD UK; 3 Department of Oncology and Metabolism, Academic Unit of Reproductive & Developmental Medicine, 4th Floor Jessop Wing, Tree Root Walk, Sheffield, S102SF UK

**Keywords:** Circadian, Fetal heart rate, Fetal heart rate monitoring, Electronic fetal monitoring, Fetal electrocardiogram, Fetal sex, Pregnancy

## Abstract

**Background:**

Previous researchers have studied circadian changes in the fetal heart rate (FHR) on small sample sizes and in a strictly controlled environment. This study was undertaken to investigate these changes during the late second and third trimesters, using a portable fetal electrocardiogram recording device (Monica AN24) in pregnant women in home and hospital environments with unrestricted mobility.

**Methods:**

This was a prospective cohort study of 54 pregnant women with uncomplicated singleton pregnancies between 25 and 40 weeks gestation. FHR recordings were made up to 16 h at home or in the hospital setting in the United Kingdom. FHR data over 90 min periods were averaged and the day (7:00 am–11:00 pm) and night (11:00 pm–7:00 am) data from the same individual were compared. Data were examined for evidence of sex-related differences.

**Results:**

During the night, there was a significant reduction in basal heart rate (bFHR) and a significant increase in short term variation (STV) and long term variation (LTV) (*P* < 0.05). Basal FHR decreased (*P* < 0.002), whereas LTV increased (*P* = 0.014) with advancing gestation. Male fetuses showed greater day: night variation than females regardless of gestation (*P* = 0.014). There was a higher bFHR in fetuses monitored during the day in hospital (*P* = 0.04).

**Conclusion:**

This study demonstrates that there are sex-, environment and time-related differences in the FHR parameters measured. These differences may need to be considered taken when interpreting FHR data.

## Background

The presence of a circadian pattern in fetal heart rate (FHR) [1, 2] and activity [3] is well documented. However, the origin of this variation is uncertain. Animal work has confirmed that in the adult information on circadian variation, which is driven by the biological clock located in the suprachiasmatic nuclei of the hypothalamus, is conveyed to the heart via the autonomic nervous system (ANS) [4]. It is less clear whether the fetal suprachiasmatic nucleus plays such a role in the generation of prenatal diurnal patterns, particularly as the fetus is influenced by the mother’s rhythm from late mid-trimester [5]. Whilst circadian rhythms are affected by daylight exposure, the fetus is not directly exposed to light but still retains a clear circadian pattern.

Circadian variation in FHR in the human fetus has been reported from as early as 20–22 weeks through to the end of pregnancy [1]. The fetal suprachiasmatic nucleus can be clearly identified from 23 weeks gestation [6] and it therefore seems logical that fetal circadian patterns should be discernible from early in the mid-trimester. FHR variability increases markedly with gestational age [7], suggesting development of fetal ANS. In addition, fetal behavioural state and maturation [8] fetal rest-activity cycles [9] and general body and breathing movements can also influence FHR and its variability [10].

It is unclear to what extent the fetus is influenced by the maternal rhythm. Studies from 26 weeks gestation to term have suggested that there is a direct relationship between the mother’s and fetal heart rates, implying that the fetus is not autonomous in this respect [5, 2]. A twin pregnancy discordant for anencephaly did not show any correlation in heart rate patterns with maternal activity, particularly in the anencephalic twin suggesting that an intact fetal central nervous system is required for the circadian rhythm to be evident [11].

Differences in heart rate variability (HRV) with respect to sex have been reported in fetuses [12], adults [13] and both pre-pubertal children and fetuses, with boys having a higher HRV than girls [14, 15].

Previous studies on circadian patterns of FHR variables including basal fetal heart rate (bFHR), short and long term variation (STV, LTV), and high variation (HV) have been conducted in hospital under laboratory conditions with controlled maternal activity (including diet) and most have studied small numbers [1, 3, 5, 16].

The introduction of a small, portable fetal electrocardiogram (ECG) recording device capable of continuous recording for up to 16 h (Monica AN24) enabled us to examine changes in the day and night FHR patterns while the women continued their-day-today activities. This investigation was undertaken in home and hospital environments to assess if place of monitoring affected FHR patterns.

## Methods

This was an observational prospective cohort study, which was approved by the local Research Ethics Committee (REC reference number: 07/Q0108/127). All eligible women over 20 weeks gestation, attending maternity assessment unit, antenatal clinic and antenatal wards at City Campus, Nottingham University Hospitals NHS Trust, Nottingham, UK were invited to participate.

Fifty-four pregnant women (28 women with gestational age (GA) ≤ 34 completed weeks and 26 with GA >34 weeks) with uncomplicated singleton pregnancies consented and participated in the study. GA varied between 25 and 40 weeks (median GA was 34 weeks). At the time of recording, none of the women used tobacco, alcohol or received any medication.

Exclusion criteria were known fetal malformation, fetal growth restriction, maternal hypertension, inability to provide informed consent, maternal smoking or alcohol dependence, in active labour, multiple pregnancies and pre-existing medical conditions such as diabetes, thyroid and cardiac disease. Inclusion criteria were a singleton pregnancy >24 weeks gestation confirmed by early ultrasound scan and maternal age at least 18 years and above. After giving informed written consent, women were recruited for a single recording, using the Monica AN24, to be made either at home (*n* = 30) or during admission to the antenatal ward (*n* = 24) starting between 13.00 and 16.00 h and lasting for up to 16 h. All women recruited for the study were given instruction on how to turn the monitor off and, if they had home monitoring, returned it to the hospital at their convenience. Women were requested to fill in a questionnaire to identify any concerns and to record their views on the comfort of the monitoring session.

Fetal electrophysiological data recorded within the monitor were downloaded via a USB connection. The methodology used for FHR signal extraction and analysis has been described in detail by others before [17]. The software programme installed in the device not only extracts the traditional FHR data obtained from conventional Doppler-based CTG monitors, it also generates beat-to-beat information on FHR providing a deeper insight into the understanding of complex development of FHR and its variation. In addition, the device also records the FHR parameters which are part of Dawes and Redman analysis [17–19].

In order to investigate changes in FHR pattern between the day and night times, three consecutive 30 min frames with FHR acquisition success rate of ≥80 % were selected randomly during the “day” (07.00–23.00) and “night” (23.00–07.00) periods for each subject.

The FHR parameters studied from the Dawes Redman analysis in this paper were:

- bFHR: basal FHR or resting level of the FHR once accelerations and decelerations have subsided,
- number of accelerations:,defined as a rise in FHR above the baseline greater than 10 bpm and lasting for more than 15 s
- STV: Short Term Variation. Computed from any minute which does not contain a deceleration or part of a deceleration and does not have a high signal loss (>10 %). For each valid minute, the STV is calculated as the average difference of adjacent 3.75 s epochs of FHR
- LTV: Long Term Variation or sometimes referred to as Mean Minute range. In any valid minute (i.e. no deceleration or large signal loss) this is the difference between the largest 3.75 s FHR and the smallest 3.75 s FHR and is expressed in milliseconds (ms)
- HV: High Variation. This is a section of the 3.75 s FHR trace where the minute range is above 32 ms for 5 out of 6 consecutive minutes.
- RMSSD: Root Mean Square of Successive Differences. This is – a measure of true beat to beat variability and is calculated from the beat to beat fetal ECG (fECG) data and not the average 3.75 s FHR (hence strictly it is not part of the traditional FHR Dawes Redman extracted parameters). The RR interval is determined for each pair of (fECG) beats and the difference is determined between consecutive RR intervals. From each 1 min of RR interval differences a root mean square (RMS) value is calculated in milliseconds (ms).

The above FHR parameters were averaged over the three 30 min epochs and compared between the day and night of the same individual.

### Statistical analysis

Kolmogorov Smirnoff analysis was first used to test for normality of the FHR data. Most FHR parameters followed a normal distribution; therefore parametric testing was used for statistical analysis. Independent sample *t* test was used to compare the demographics between the recordings made at home and in hospital. In order to analyse the day and night recording of the same individual, the paired sample *t*-Test was used for bFHR, STV, LTV and RMSSD or the Wilcoxon paired sample test for accelerations and HV. Univariate analysis of variance was considered to measure the association of FHR parameters with time and place of recording. Correlation between FHR parameters and gestational age was assessed using Pearson’s rank-order correlation coefficient.

## Results

Table 1 summarises the basic demographic data for the women and their fetuses monitored at home and in hospital.

**Table 1:** Basal demographic data for the women and fetuses studied at home and in hospital setting

Variables	Home setting (*n* = 30)	Hospital setting (*n* = 24)	*P* value
Age (y)	28.9 ± 5.6	28.1 ± 7.1	*P* = 0.7
Body mass index (kg/m^2^)	26.3 ± 5.7	27.5 ± 5.8	*P* = 0.5
Gestational age at recording (completed weeks)	33.8 ± 3.9	34.2 ± 3.0	*P* = 0.5
Gestational age at delivery (days)	273 ± 17	273 ± 17	*P* = 0.7
Birth weight of the babies (kg)	3.46 ± 0.55	3.48 ± 0.53	*P* = 0.9
Birth weight of the babies (centiles)	57 [38–74][30]	47 [34–77]	*P* > 0.5

Data are shown as mean ± SD or median [interquartile]

One hundred eight recordings (54 day and 54 night) made over 90 min epochs were analysed from 30 women recruited at home and 24 at hospital (Table 2). A significant reduction in basal heart rate and a significant increase in STV and LTV was observed in all fetuses at night, whether monitored at home or in hospital. Although all fetuses exhibited an increased number of accelerations at night, statistically significant results were observed only for the fetuses recruited at home (*P* < 0.001 home; *P* > 0.1 hospital)).

**Table 2:** Basic comparison of day and night fetal heart rate (FHR) parameters in normal fetuses at home and in hospital setting

	Home (*n* = 30)			Hospital (*n* = 24)		
Parameters	Day recordings	Night recordings	*P* value	Day recordings	Night recordings	*P* value
bFHR (bpm)	136.1 ± 9.2	131.6 ± 7.4	=0.005	142.6 ± 9.3	131.9 ± 8.6	<0.0001
STV (ms)	10.9 ± 2.7	11.8 ± 2.8	0.04	10.5 ± 2.7	11.7 ± 3.1	=0.024
LTV (ms)	58.3 ± 14.1	63.3 ± 14.8	=0.009	56.3 ± 14.2	63.4 ± 16.6	=0.017
Accelerations (numbers)	10.0 [6.5–14.0]	13.0 [9.8–17.0]	<0.0001	10.2 [7.0–13.8]	13.5 [9.0–17.0]	>0.1
HV (ms)	58.5 [43.3–68.3]	58.5 [51.4–73.5]	=0.09	51.7 [37.1–66.7]	67.2 [54.1–72.8]	<0.001
RMSSD (ms)	11.0 ± 1.6	10.7 ± 1.6	>0.3	10.7	10.3	>0.5

Data are shown as mean ± SD or median [IQR]

*bFHR* basal fetal heart rate, *STV* short term variation, *ms* milliseconds, *LTV* long term variation, *HV* high variation, *RMSSD* root mean square of successive difference

**Fig. 1:**
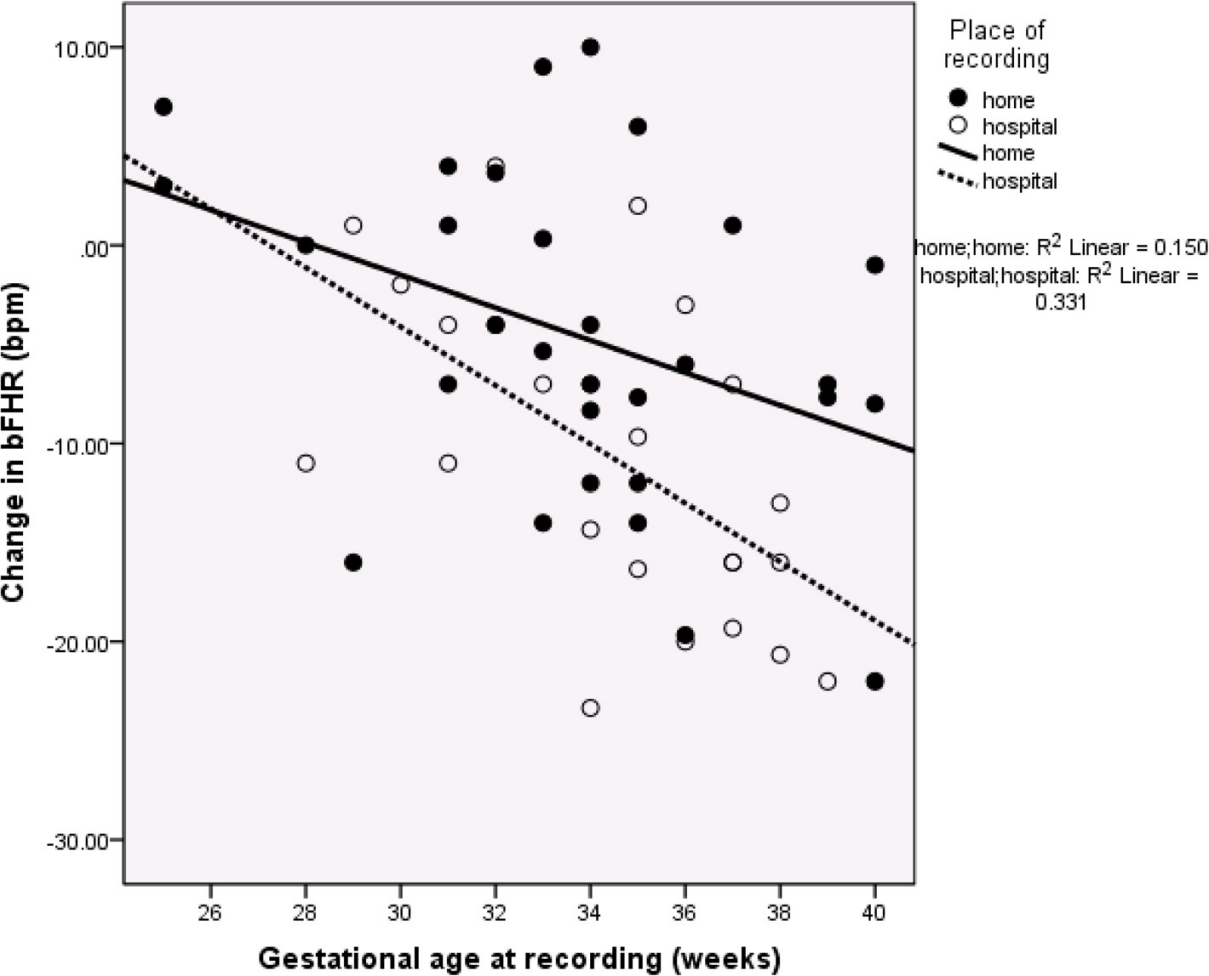
Analysis of Day: Night Changes in Basal Fetal Heart Rate. The day:night change in basal fetal heart rate (bFHR) increased significantly with gestation age at the time of recording (*r* = −0.409; *P* < 0.002). The place of recording also exerted an additive significant effect (final *r* = −0.554; *P* < 0.001)

Having demonstrated a significant day: night effect, we calculated the day: night difference for each measurement, to allow further analysis in relation to absolute difference. Regression analysis showed the change in bFHR to be strongly reciprocally-associated with GA at the time of recording (Fig. 1; *P* < 0.002); the addition of place of recording to the analysis significantly (*P* < 0.01) improved the final r to 0.554 (*P* < 0.001). The change in LTV was directly related to GA (*r* = 0.331; *P* = 0.014); there was no effect of GA at recording or place of recording on day: night change in either STV or RMSSD (*P* > 0.1, >0.3 respectively).

In our study, 30 fetuses were male and 24 female. Although bFHR was very similar in male and female fetuses (day: 139.2 ± 7.4 and 138.8 ± 12.2 bpm; night 131.2 ± 6.4 and 132.4 ± 9.4 bpm), males had significantly higher night-time STV and RMSSD than did females (12.5 ± 3.0 compared with 10.6 ± 2.7 ms, and 11.0 ± 1.5 compared with 9.7 ± 1.8 ms; *P* = 0.014, *P* = 0.004 respectively); the sex-related difference also approached significance for LTV (*P* = 0.057). There were no sex-associated differences during the daytime recordings.

**Fig. 2:**
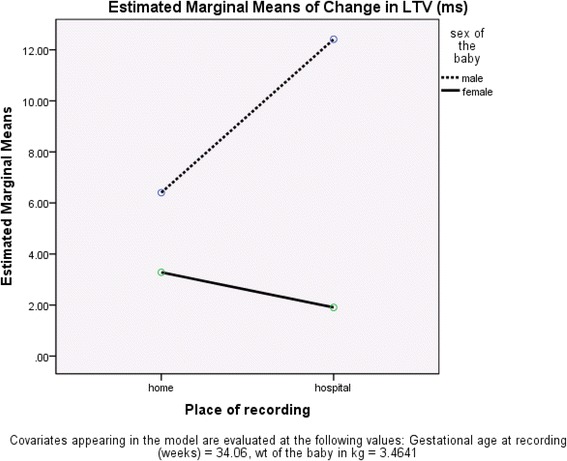
Analysis of Day: Night Changes in Long Term Variation. Analysis of the day:night change in long-term variation (LTV) showed significant effects of both gestational age (GA) at recording (*P* = 0.030) and sex (*P* = 0.029); birthweight (used as a surrogate for weight at recording) did not have a significant additional effect (*P* > 0.1)

Table 3 shows the day-night differences with respect to sex. This difference was significant for LTV (*P* = 0.01) and remained significant when GA at recording was included in the analysis (Fig. 2), and was independent of birth weight.

**Table 3:** Day: night differences in fetal heart rate (FHR) parameters with respect to sex

	Male (*n* = 30)	Female (*n* = 24)	*P* value
Change in bFHR (bpm)	−7.3 [−3.3, −14.0]	−5.7 [+1.0, −15.6]	>0.4
Change in STV (ms)	1.2 [3.1, 0.2]	0.6 [1.6, −1.1]	0.076
Change in LTV (ms)	7.7 [17.6, 5.0]	3.5 [7.5, −5.6]	0.010
Change in RMSSD (ms)	0.0 [1.3 -1.3]	−0.31 [0.4, −1.5]	>0.2

Data are shown as mean median [IQR]

The day: night change in all measures of FHR variability was greater in male than female fetuses; this difference was significant with respect to long-term variation (LTV)

*bFHR* basal fetal heart rate, *STV* short term variation, *LTV* long term variation, *RMSSD* root mean square of successive differences

When place of recording was taken as a fixed factor in our univariate model of analysis, a significantly higher bFHR (*P* = 0.04) was observed in the day time (142.5 ± 9.3 bpm) for the recordings made in hospital compared to the home setting (136.1 ± 9.2 bpm). None of the other normally-distributed parameters showed any significant difference when place of the recording was taken into account as well as sex.

## Discussion

This study has demonstrated that there is a clear circadian pattern of changes in the FHR, with a greater influence of time on bFHR and LTV with advancing gestation. Male fetuses show a greater difference than females in STV when day and night recordings are compared, regardless of gestation. The bFHR is higher in fetuses monitored in hospital than those monitored in the home.

Maternal activity, diet intake, psychological and physiological conditions can influence FHR [8, 20, 21]. The fact that information regarding these maternal factors were not collected can be considered as a limitation of this study. However, the ability to achieve good quality recordings of the FHR over prolonged periods (up to 16 h) whilst the mother continued with normal activities at home is a novel approach, permitting the assessment of the FHR under normal (uncontrolled) conditions. This is the first study to report changes seen in the FHR in relation to time of day in pregnancies where maternal mobility was not restricted.

Because we did not investigate the relationship of the FHR to that of the mother, it is not possible to comment on whether the control of the circadian pattern in the fetus is partially under fetal control or partially or totally under maternal influence. However, the fact that advancing GA has a clear influence on the size of the day: night differences in both bFHR and LTV supports the theory that there is a major fetal component. This is supported by a small study looking at nine pregnancies at term that did not demonstrate a significant relationship between maternal and fetal heart rates [21]. In addition, although this study did not collect data as regards the quality of the mothers sleep, a prospective trial on the effect of obstructive sleep apnea in pregnancy on fetal heart rate monitoring found there was no effect [22].

In order to assess day: night changes in FHR pattern, we randomly selected the best 90 min FHR data with good fetal signals from the day and night recording of the same individual. Therefore we recognise that our study suffered from selection bias. However, selection of 90 min data FHR data guaranteed the analysis of high variation episodes as in human fetus the cycle of active and quiet sleep ranges from 60–90 min [23]. Nonetheless, our results are in keeping with previous studies, all performed under strictly controlled laboratory conditions, confirming the presence of a circadian pattern in the FHR. The night time reduction in bFHR is also in keeping with previously published data [5, 24]. Not all researchers have confirmed the increase in measures of variability during the night that we have described, but some have been small studies using visual rather than computerised analysis of the heart rate recordings [25]. The increase in variability is in keeping with the known increase in fetal activity seen at night [3]. However, there are also longer rest phases seen at night [23] which could, at least in part, explain the lower bFHR.

To date, few studies have evaluated sex-related FHR differences and most of them have failed to demonstrate significant difference in the antepartum period. It has been suggested that sex does not need to be considered when assessing FHR variability [26], this was based on a small study looking at 5 min recordings of FHR whereas our data were obtained from recordings over three hours (90 min day and 90 min night). Both Fleisher et al. [27], in a longitudinal study of 14 male and 17 female fetuses studied over 15 min periods and Amorim-Costa et al. [12], in a cross-sectional retrospective study of 5172 male and 4529 female fetuses studied for an average duration of 30 min, reported significant sex-related differences in FHR parameters at various gestational ages. Baseline FHR was consistently found to be higher in female fetuses whereas STV and LTV tend to be lower at most stages of pregnancy. Our data confirm greater heart rate variability, expressed as several indices, in male than female fetuses. This adds to existing knowledge by showing that this increased variability in males is significant at night (when bFHR is lower and maternal conditions are at their most stable) but not during the day. Possible mechanisms for this sex-related difference seem not to have been addressed. Animal work has shown that female fetuses have significantly greater adrenomedullary mRNA expression of catecholamine-synthesizing enzymes than males [28]. The greater heart rate variability in male fetuses suggests the possibility of increased sensitivity to circulating catecholamine. Sex-related differences in catecholamine receptor density or affinity have not yet been investigated.

## Conclusions

The existence of circadian rhythms has been demonstrated in most living organisms including plants and some bacteria. This study confirms that the human fetus demonstrates a clear circadian pattern in both the underlying heart rate and other variables. It is probable that there is, at least, a large fetal component to the control of this pattern. The demonstration of sex-related differences, particularly in terms of the size of the differences seen between day and night, supports this.

The changes in both fetal activity and heart rate variables with the time of day [29] have implications for the assessment of fetal well-being. Knowledge of the circadian pattern of fetal activity and heart rate will improve the interpretation of fetal monitoring.

## Abbreviations

ANS: Autonomic nervous system
bFHR: Basal fetal heart rate
ECG: Electrocardiogram
Fetal ECG: fECG
FHR: Fetal heart rate
GA: Gestational age
HRV: Heart rate variability
HV: High-variation
LTV: Long-term variation
ms: Millisecond
RMS: Root-mean square
RMSSD: Root mean square of successive difference
STV: Short-term variation
